# Restoration of Sensitivity in Chemo — Resistant Glioma Cells by Cold Atmospheric Plasma

**DOI:** 10.1371/journal.pone.0064498

**Published:** 2013-05-21

**Authors:** Julia Köritzer, Veronika Boxhammer, Andrea Schäfer, Tetsuji Shimizu, Tobias G. Klämpfl, Yang-Fang Li, Christian Welz, Sabina Schwenk-Zieger, Gregor E. Morfill, Julia L. Zimmermann, Jürgen Schlegel

**Affiliations:** 1 Max Planck Institute for Extraterrestrial Physics, Garching, Germany; 2 Department of Neuropathology, Technical University Munich, Munich, Germany; 3 Department of Head and Neck Cancer, Ludwig Maximilians University, Munich, Germany; Johns Hopkins Hospital, United States of America

## Abstract

Glioblastoma (GBM) is the most common and aggressive brain tumor in adults. Despite multimodal treatments including surgery, chemotherapy and radiotherapy the prognosis remains poor and relapse occurs regularly. The alkylating agent temozolomide (TMZ) has been shown to improve the overall survival in patients with malignant gliomas, especially in tumors with methylated promoter of the O6-methylguanine-DNA-methyltransferase (MGMT) gene. However, intrinsic and acquired resistance towards TMZ makes it crucial to find new therapeutic strategies aimed at improving the prognosis of patients suffering from malignant gliomas. Cold atmospheric plasma is a new auspicious candidate in cancer treatment. In the present study we demonstrate the anti-cancer properties of different dosages of cold atmospheric plasma (CAP) both in TMZ-sensitive and TMZ-resistant cells by proliferation assay, immunoblotting, cell cycle analysis, and clonogenicity assay. Importantly, CAP treatment restored the responsiveness of resistant glioma cells towards TMZ therapy. Concomitant treatment with CAP and TMZ led to inhibition of cell growth and cell cycle arrest, thus CAP might be a promising candidate for combination therapy especially for patients suffering from GBMs showing an unfavorable MGMT status and TMZ resistance.

## Introduction

Glioblastoma (GBM) is the most common and lethal primary brain tumor in adults and is classified according to the world health organization (WHO) as a grade IV tumor. These tumors are a highly invasive, rapidly spreading form of central nervous system cancer which are resistant to surgical and medical treatment. Particular challenges of treating GBM are its distinct tumor heterogeneity, the inability of treatments to reach all tumor cells, the delivery of drugs across the blood-brain barrier and the high likelihood of relapse, which is often rapid and aggressive. Although some advances have been made in recent years, treatment remains palliative for most patients as a cure remains elusive. Looking at the numbers the median survival has improved from 12.1 to 14.6 months, but still less than 16% of the patients survive three years postdiagnosis [1].

The first-line chemotherapeutic drug is the alkylating agent temozolomide (TMZ). Following oral absorption, TMZ is converted to an alkylating methyldiazonium cation that is known to damage DNA thereby leading to DNA double strand breaks [2],[3]. The enzyme O6-methylguanine-DNA methyltransferase (MGMT) is capable of counteracting the cytotoxicity induced by TMZ [4],[5] - thus tumors expressing high levels of MGMT (MGMT positive, unfavorable) are more resistant to TMZ than those in which the enzyme has become silenced by promoter methylation (MGMT negative, favorable). MGMT promoter methylation is associated with a favorable outcome and predicts a benefit from alkylating agent chemotherapy in patients with newly diagnosed glioblastoma [6],[7],[8],[9]. In a large randomized multicenter trial an unmethylated MGMT promoter (protein is expressed - unfavorable MGMT status) was observed in more than half of the patients and those therefore did not benefit from the TMZ treatment [10]. Thus, there is a clinical need to establish additional novel therapy regimes to overcome TMZ resistance. Therefore in the present study the concomitant treatment of GBM with cold atmospheric plasma (CAP) and TMZ in overcoming TMZ resistance was investigated.

In the past years CAP – a partially ionized gas - proved its effectiveness for different applications in health care and medicine. In a combined effort of physicists, engineers, chemists, biologists and medical doctors several different CAP sources were developed, characterized and to some extent optimized for their respective application. All these plasma sources have in common that they generate CAP thereby initiating reactions in the surrounding air, which lead to the production of a reactive mix of electrons, ions, neutrals, reactive species and UV light. Nevertheless depending on the plasma source properties, composition and concentrations of the produced species can be varied and therefore initiate different reactions with the respective target.

Several developed CAP sources have proven to successfully inactivate bacteria, fungi, virus and spores in a dose-dependent manner [11],[12],[13],[14],[15]. Healthcare applications such as the sterilization of surgical instruments [16],[17],[18], skin [19],[20] and wound disinfection [21],[22] therefore paved its way into medical care. Further generations of CAP sources however also showed anti-cancer properties. Main targets for CAP in cancer cell lines were growth inhibition [23], inhibition of cell migration and invasion (in colorectal cancer cells [24]) or induction of apoptosis (in melanoma cells [25], [26], mouse lung carcinoma [27]). The production of reactive oxygen and nitrogen species by CAP is thought to be a key player initiating the anti-cancer properties of CAP. It was shown that intracellular ROS levels increase after CAP treatment, thereby inducing DNA damage and apoptosis in the cells [28], [25], [29], [30].

In this study we show that TMZ resistant cells with an unfavorable MGMT status as well as TMZ sensitive cell lines are susceptible to CAP treatment. Furthermore we are able to demonstrate a synergistic effect of a combined treatment with TMZ and CAP in cells with unfavorable MGMT status.

## Materials and Methods

### Plasma Device

**Figure 1:**
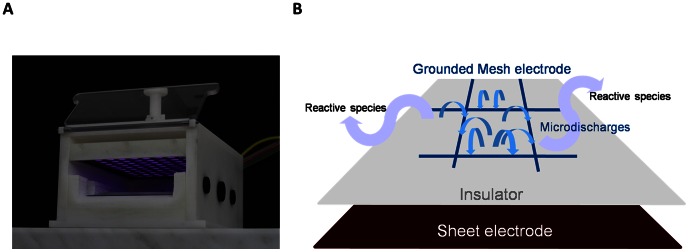
The CAP device based on Surface Micro Discharge technology. **A** Photo of the FlatPlaSter 2.0 during plasma production. **B** Sketch of the plasma production on a Surface Micro-Discharge (SMD) electrode. The plasma discharge is ignited on the SMD electrode and the reactive species are produced in the plasma by electrons from air molecules. The produced reactive species reach the sample by diffusion and induce there biological reactions.

The CAP device employed in this study uses the Surface Micro Discharge (SMD) technology for plasma production as shown in figure 1 and has been published and characterized in detail in Morfill et al. [11] and Maisch et al. [12]. In short, the electrode for plasma production is located at the top inside a closed box (figure 1 A). The electrode consists of a Teflon (insulator) plate sandwiched by a planar brass plate (sheet electrode) and a stainless steel mesh grid (figure 1 B). Applying high sinusoidal voltage of 8.5 kV_pp_ with a frequency of 1 kHz, micro-discharges are generated homogenously across the mesh grid side of the sandwich in the ambient air. The power consumption for the plasma discharge is ∼10 mW/cm^2^ and was measured with the Lissajous method using a 1 µF capacitance [31]. The glioma cells were treated in 6 or 96-well plates which were placed directly beneath the electrode. The distance between the sample and the electrode was set to 14 mm. As already mentioned in the introduction, the plasma produces electrons, charged particles, reactive species (mainly reactive oxygen and nitrogen species), UV light and heat. The main constituents produced by the CAP device used in this study were measured and have been summarized in Maisch et al. [12]. In short, for the treatment times used in this study, the temperature increase was measured to 4 degrees above the ambient temperature at maximum. The main UV components emitted by the device are in the wavelength range between 280 and 400 nm. Furthermore, negligible intensities of UVC light emission are detected. The UV power density was measured to be 25 nW/cm^2^. Concerning the production of reactive species of the device mean values of approximately 500 ppm for O_3_, <1 ppm for NO and 3 ppm for NO_2_ were measured at the end of the application. In our study, mainly the produced reactive species are transported to the samples and trigger the biological reactions. There are almost no electrons and ions due to the distance between the electrode and samples of 14 mm.

### Cell culture and cell viability

The human glioblastoma cell lines LN18, LN229 and U87MG were purchased from American Type Culture Collection (ATCC, Rockville, MD, USA) and routinely cultured in Dulbecco's modified Eagle's serum GlutaMAX™ (DMEM GlutaMAX™, Invitrogen) supplemented with 10% fetal calf serum (FCS) and 100 U/ml penicillin and 100 µg/ml streptomycin (Biochrom AG, Berlin, Germany) under standard cell culture conditions at 37°C and 5% CO_2_.

For the investigation of cell proliferation after the treatment with cold atmospheric plasma (CAP) and/or temozolomide (TMZ), 5×10^3^ cells were seeded on a 96-well plate and grown over night until they reached 80% confluence. Cells were treated without medium covering them and fresh medium (DMEM) with 1% FCS was added immediately afterwards. TMZ treatment (50 µM, 100 µM and 200 µM), if applied in a combined therapy, was carried out immediately after a single CAP treatment. Medium was changed every 24 h and fresh TMZ was added for three days consecutively. Controls were kept without medium for the same duration as CAP treatment and/or were DMSO treated in equal concentrations as TMZ. Cells were incubated for 48 h at 37°C and 5% CO_2_ before analyzing the cell viability using the Cell Proliferation Kit I (MTT assay, Roche, Basel, Switzerland). The color change was quantitated at 595 nm using a scanning multi-well spectrophotometer.

The above described experiments were performed in six replicates and data were expressed as the mean of the replicate determinations (X ± SEM) in percent of absorbance of samples with untreated cells (100%).

### Immunoblotting

Protein lysates were taken at the indicated time points after CAP treatment. 20 µg of protein were separated by 12% SDS-PAGE and transferred to PVDF membranes (Millipore, Billerica, MA, USA), which were incubated with primary antibodies (MGMT 1∶2500; yH2AX 1∶2500; PARP1 and cleaved PARP1 1∶ 2000; all antibodies from CST, Danvers, MA, USA; GAPDH, 1∶50000, Sigma Aldrich, Hamburg, Germany) over night at 4°C followed by horseradish peroxidase-labeled secondary antibodies (1∶10000; CST, Danvers, MA,USA) for 1 h at RT. Signals were visualized by ECL Western Blotting Detection (Millipore, Billerica, MA, USA).

### Clonogenicity assay

Cells were seeded in 6 cm dishes and treated with CAP for 0, 30, 60 and 120 seconds without medium and/or with TMZ (50 µM, 100 µM, and 200 µM) afterwards. As described earlier fresh medium was added immediately after the CAP treatment. Controls were treated equally. Cells were seeded 24 h after the CAP treatment into 6-well plates and allowed to form colonies over a time period of 12 days. Fixation and staining of the colonies was performed using the DiffQuik Kit (Medion Diagnostics, Düdingen, Switzerland). Colonies of more than 50 cells were counted. The experiment was repeated three times for each cell line.

### Flow cytometry

Cell cycle analysis was carried out by flow cytometry. Cells were seeded in 100 mm^2^ tissue culture dishes (1×10^6^ cells/dish), allowed to attach overnight, and CAP treated and/or TMZ treated for the indicated times and indicated concentrations. Controls were treated equally. For FACS analysis cells were washed twice in phosphate-buffered saline (PBS) and fixed in ice-cold 70% methanol at 4°C for at least 2 h. Afterwards cells were washed with PBS and then incubated with 100 µg/ml of RNase A (Sigma-Aldrich, Hamburg, Germany) for 20 min at 37°C and stained with Propidium iodide (PI, 50 µg/ml). Cell cycle distribution was analyzed using the BD FACSCalibur (Becton and Dickinson, Heidelberg, Germany) and FlowJo Software (Flowjo, Cincinnati, OH, USA).

### Statistical analysis

All statistical significances were evaluated using one or two factor analysis of variance (ANOVA). Differences were considered significant at p<0.001.

## Results

**Figure 2:**
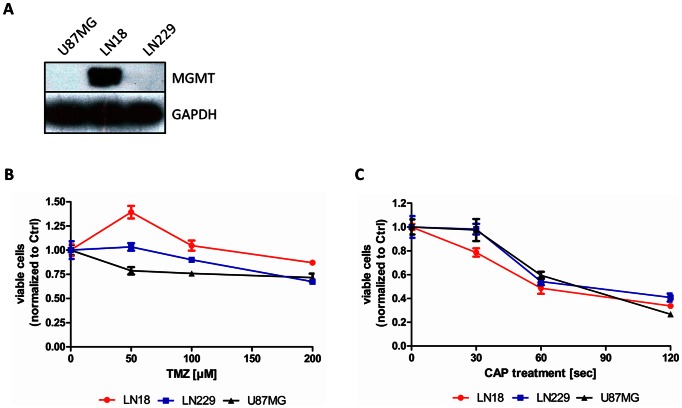
Inhibition of proliferation by TMZ or CAP treatment. **A** Immunoblotting of the investigated cell lines for expression of the MGMT protein. Lysates of U87MG, LN18 and LN229 cells were blotted against the anti-MGMT antibody, GAPDH served as a loading control. MGMT protein is expressed in the LN18 cell line, U87MG and LN229 cells do not express the MGMT protein. **B** U87MG, LN229 and LN18 glioma cells were treated with 50 µM, 100 µM and 200 µM TMZ for three days consecutively. The number of viable cells was measured using the MTT assay after the treatment with TMZ. Statistical significances are evaluated for 200 µM of TMZ in the LN229 and U87MG cell lines. P-value: 0.001 **C** The same cell lines were treated with CAP in a 96-well plate without medium. Fresh medium was added to the cells immediately after the CAP treatment. Cell growth was measured 48 h after CAP treatment. Statistical significances were observed after 60 seconds of treatment in all tested cell lines. P-value *** <0.001.

In this study the effects of cold atmospheric plasma (CAP) on different glioma cell lines were analyzed in detail. For this purpose U87MG, LN229 (both MGMT negative, favorable status) and LN18 (MGMT positive, unfavorable status) cells were treated with a CAP (produced by the Surface Micro Discharge technology). The status of the MGMT protein was confirmed by immunoblotting of cell lysates against anti-MGMT antibody. The cell lines U87MG and LN229 do not express the MGMT protein, whereas the cell line LN18 highly expresses the MGMT protein under normal culturing conditions (fig. 2 A).

### Dose dependent inhibition of glioma cell growth after CAP treatment

Preliminary control experiments using the cell lines U87MG, LN229 and LN18 showed that they are able to survive handling with only little medium covering them for at least 5 minutes (data not shown). This resembles the situation in the patient during resection, at the time point when CAP could be applied as a single treatment to the resection cavity.

In this study the cells were therefore treated once for 30, 60 and 120 seconds without medium. The medium was removed before treatment thus remaining only a thin film of liquid covering the cells. Immediately after the single CAP treatment fresh medium was added to the cells.

Using the MTT assay the effect of different CAP exposures on LN18, LN229 and U87MG glioblastoma cell proliferation was investigated. Therefore the cells were seeded in 96-well plates for 24 h and afterwards were treated with CAP as described above (fig. 2 C). For comparison the glioblastoma cell proliferation was also investigated after three days of consecutive treatment with the chemotherapeutic TMZ (fig. 2 B). A dose dependent inhibition of cell growth after CAP treatment for 30 to 120 seconds was observed for all treated cell lines, including the TMZ resistant cell line. A significant inhibition of about 45 percent was observed after 60 seconds of CAP treatment and a notably reduction of about 65 percent after 120 seconds of CAP. Unlike the CAP treatment, repeated treatment with TMZ in concentrations of up to 200 µM for three days consecutively resulted in reduced viability of only 25 percent for the MGMT negative (favorable MGMT status) cell lines LN229 and U87MG. Treatment of the MGMT positive (unfavorable MGMT status) cell line LN18 led to an induction of cell growth when treated with low concentrations of TMZ (50 µM). Administration of higher concentrations of TMZ (up to 200 µM) showed no significant inhibition of proliferation (fig. 2 B).

### Induction of DNA damage by CAP

**Figure 3:**
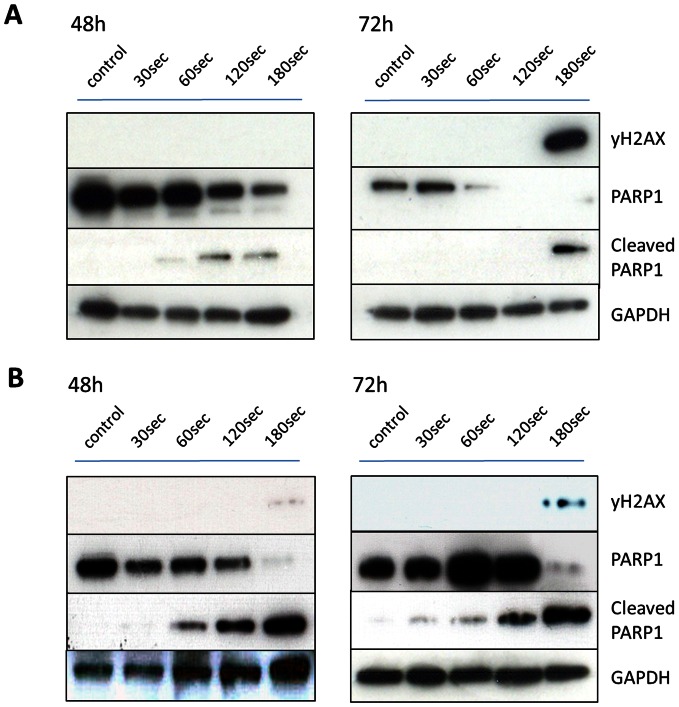
Induction of DNA damage. **A** Representative immunoblotting of LN229 cells (MGMT negative) and staining for cleaved PARP1, PARP1 and yH2AX as a marker for DNA damage was performed 48 h and 72 h after CAP treatment. GAPDH served as the loading control. **B** Similar results were observed 48 h and 72 h after the treatment for the MGMT positive cell line LN18.

The discovered growth inhibition after the CAP treatment was likely conducted by DNA damage, which was observed by immunoblotting with antibodies against cleavage of PARP1 and yH2AX (fig. 3). Protein samples were taken 48 h and 72 h after the respective CAP treatment (without medium). GAPDH served as a loading control.

The results show that for CAP exposures of 60 seconds and longer in LN229 and LN18 cells exhibit PARP1 cleavage 48 h post treatment. However, induction of yH2AX was not detected until 72 h after the treatment and not for exposure times less than 180 seconds. The phosphorylation of H2AX was less prominent in the LN18 cells than in the LN229 cells, but likewise detectable 72 h after CAP exposure for 180 seconds. Cleavage of Caspase-3 and Caspase-7 was not observed for both cell lines, even not in lysates taken 4 h and 24 h after CAP treatment (data not shown). CAP treatment for up to three minutes did not feature cytolysis or damage of the cell membrane measured by the release of lactate dehydrogenase (LDH) 2 h, 24 h and 48 h after treatment in LN18 cells. An increase in released LDH was measured 48 h after CAP treatment for 10 minutes (Figure S1). Furthermore, DNA damage at the level of single cells was observable to a minor content by the comet assay 1 h after CAP in LN229 cells, but was repaired 24 h after the treatment (Figure S2).

### Cytostatic effect induced by CAP treatment in glioma cells

**Figure 4:**
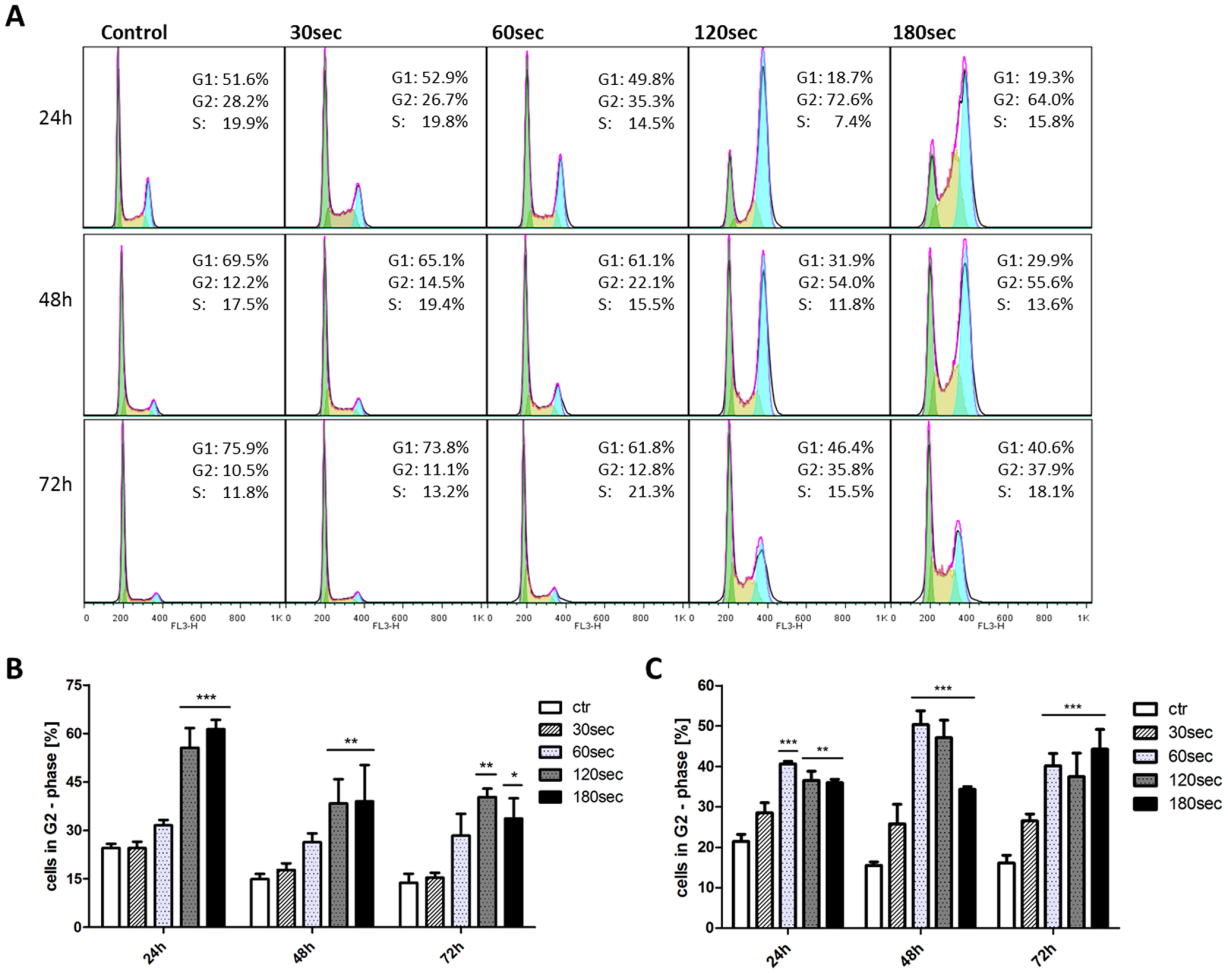
Cell cycle arrest in G2/M-phase. **A** Cell cycle analysis of U87MG cells was performed 24 h, 48 h and 72 h after CAP treatment (30 s, 60 s, 120 s and 180 s) by flow cytometry. Treatment was performed only with a thin film of liquid covering the cells. Similar results were observed for the LN229 (MGMT negative) and LN18 (MGMT positive) cells (Figure S3 and Figure S4). **B** Statistical significances of the observed arrest in the G2/M-phase in U87MG and **C** in LN18 cells. P-value *** <0.001.

Based on our previous findings, we hypothesized that CAP treatment affects cell cycle progression in glioma cells. To test this hypothesis, we analyzed the cell cycle progression of LN18, LN229 and U87MG glioma cells 24 h, 48 h and 72 h after CAP exposure (fig. 4, Figure S3 and Figure S4). Treatment of these glioma cells with CAP for 120 seconds and 180 seconds led to a two to four times higher amount of cells in the G2/M-phase of the cell cycle compared to the untreated control. This significant arrest was observable for at least 72 h and independent of the MGMT status of the cells (fig. 4 B and C). As shown in figure 4 A, no sub-G1 population was observed in cells treated for up to 180 seconds, further confirming the cytostatic effect as more prominent than the apoptotic effect of CAP on glioma cells.

### CAP treatment reduces the clonogenicity of glioma cells

**Figure 5:**
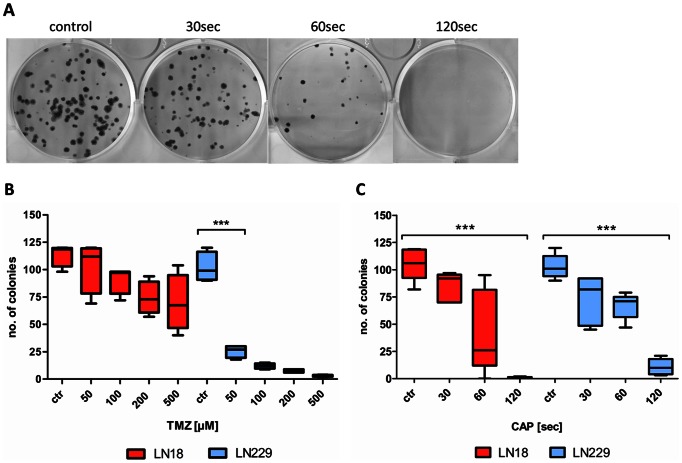
Clonogenic capacity of glioma cells treated with TMZ (B) or CAP (C). Glioma cells were either TMZ or CAP treated without medium and 24 h later 150 cells/well were seeded on a 6-well plate. Colonies formed after 12 days were stained and counted. P values *** <0.001. **A** Picture of the LN18 (MGMT positive) cells treated with CAP for 30 s, 60 s and 120 s. Afterwards the formed colonies were stained. **B** Treatment of glioma cells with TMZ with concentrations of up to 500 µM and colony formation assay was performed afterwards. **C** CAP treatment followed by the colony formation assay in either MGMT positive or MGMT negative cells.

Treatment with TMZ was able to reduce the clonogenicity in LN18 (unfavorable MGMT status) cells only to a minor content, even when treated with a concentration of 500 µM (fig. 5 B). The MGMT negative (favorable MGMT status) cell line LN229 was sensitive to treatment with 50 µM TMZ, resulting in a significant reduction of clonogenicity. Concerning the effects of CAP, we found a significantly reduced clonogenicity after treatment for both cell lines independent of their MGMT status (fig. 5 C). CAP treatment of 120 seconds furthermore culminated in a complete loss of clonogenicity in the LN18 cell line.

### Effects on cell proliferation by concomitant therapy

**Figure 6:**
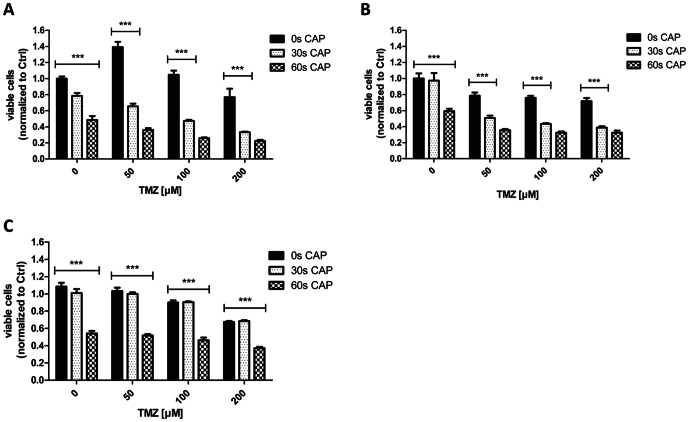
Combined treatment of glioma cells with TMZ and CAP. **A** LN18 cells were CAP treated once without medium; afterwards TMZ was applied consecutively for three days. **B** U87MG cells were CAP treated once and TMZ was applied consecutively afterwards for three days. **C** LN229 cells were CAP treated followed by TMZ treatment for three days. MTT assay for evaluating the cell viability was performed at day four. Controls were kept without medium and/or DMSO treated. P-value *** <0.001"

For these experiments the cells were CAP treated once (without medium), followed by the immediate application of TMZ (50 µM, 100 µM or 200 µM) for three days consecutively. Cell viability was measured using the MTT assay at day four (fig. 6). Combined treatment with CAP and TMZ leads to significant stronger inhibition of proliferation of U87MG cells (fig. 6 A), LN18 cells (fig. 6 B) and LN229 cells (fig. 6 C) compared to separate treatment with CAP or TMZ alone. Combined treatment with low dose of TMZ (50 µM) and short treatment of CAP (30 s in U87MG and LN18, 60 s in LN229 cells) caused significantly higher suppression of cellular growth as compared with high dose of TMZ (100 µM) alone.

### CAP treatment restores the sensitivity of TMZ resistant glioma cells

**Figure 7:**
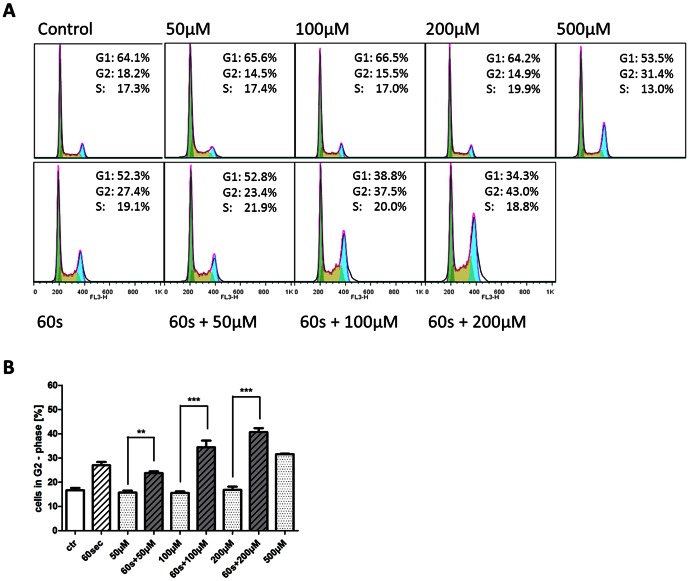
TMZ resistant cells respond with cell cycle arrest to combined treatment. **A** LN18 glioma cells were TMZ treated for three days consecutively with 100 µM, 200 µM and 500 µM and cell cycle analysis was performed afterwards. In comparison, CAP treatment without medium for 60 seconds was applied once to LN18 glioma cells, followed by TMZ treatment with 50 µM, 100 µM and 200 µM for three days consecutively. Cell cycle distribution was determined afterwards. **B** Analysis of the percentage of cells in the G2/M-phase after treatment with TMZ and combined treatment with CAP. P-value *** <0.001.

LN18 glioma cells, which are fairly resistant to repeated treatment of 500 µM with the chemotherapeutic TMZ *in vitro*, were exposed to CAP for 30 seconds up to 180 seconds. Cell viability and cell cycle distribution were determined using the MTT assay and FACS analysis. As shown earlier, CAP treated LN18 cells displayed inhibition of proliferation in a dose dependent manner (fig. 2 C), whereas treatment with TMZ even for concentrations of up to 200 µM only resulted in inhibition of proliferation to a minor degree (fig. 2 B). Furthermore, LN18 cells exhibit cell cycle arrest in G2/M-phase after one single treatment with CAP for 60 seconds or longer which was observable for at least 72 h (Figure S3). In contrast, repeated treatments of LN18 cells with TMZ with concentrations between 100 µM and 500 µM were not able to induce a comparable cell cycle arrest (fig. 7 A). Therefore, combined treatment of CAP for 60 seconds (single treatment) and TMZ (50 µM, 100 µM, 200 µM for three days consecutively) was carried out and cell cycle distribution was detected afterwards. The obtained data clearly showed that a previously applied CAP treatment restores the sensitivity of TMZ resistant glioma cells, leading to an induction of cell cycle arrest in G2/M-phase (fig. 7 B). Combined treatment revealed strong significance in inducing a cell cycle arrest, especially when 60 seconds of CAP were combined with 100 µM or 200 µM TMZ compared to treatment with TMZ alone (P-value<0.001).

## Discussion

Until today, despite intensive treatment of glioblastomas including resection, radio- and chemotherapy most GBM patients suffer from relapse. Therapy with alkylating agents like TMZ is worthwhile in a subpopulation of patients showing methylation of the MGMT gene in their tumor; however patients with unfavorable MGMT status are resistant towards therapy with TMZ [32],[33],[34],[35]. In addition, most tumors acquire resistance towards chemotherapy during treatment, accompanied by a change of the methylation status from methylated to unfavorable unmethylated MGMT status. Felsberg and colleagues speculated, that treatment with TMZ promotes survival of tumor cells with unmethylated MGMT status which give rise to resistant clones causing relapse [36]. Therefore in the present study we focused on the re-establishment of sensitivity in MGMT expressing cells towards chemotherapy by combined treatment with cold atmospheric plasma (CAP) and TMZ. CAPs have turned out to be a novel promising approach in treatment of glioma cells *in vitro* and *in vivo*.

So far, the MGMT status is the only established prognostic and predictive factor in the therapy of glioblastoma with chemotherapeutics. The MGMT status of the used cell lines therefore correlates with the resistance of the cells towards TMZ. CAP showed anti-cancer properties in chemotherapy resistant and sensitive cells, whereas TMZ was effective only in cells with favorable MGMT status. For the evaluation of anti-cancer features of CAP, treatment of cells that feature a favorable MGMT status and are highly susceptible to TMZ (LN229, U87MG), and in comparison cells that have an unfavorable MGMT status and are resistant to TMZ (LN18) (fig. 2 C) was performed. In contrast to the results obtained after repeated application of TMZ, a high reduction of viable cells was detected after the CAP treatment. Even though the observed reduction of proliferation after TMZ treatment was less pronounced than reported elsewhere, the strong reduction of clonogenicity and the induction of a cell cycle arrest in the U87MG and LN229 cells illustrated the effectiveness of TMZ in these cell lines.

We observed a dose dependent inhibition of proliferation by CAP treatment for all three cell lines – even in the LN18 cells that are resistant to TMZ (unfavorable MGMT status) treatment of up to 200 µM. Noteworthy, CAP was extremely efficient in reducing the cell viability. Induction of DNA damage after CAP treatment was confirmed by detection of markers for DNA damage (fig. 3) and by comet assay (supplements). Controversially, cleavage of caspases and cytolysis could not be observed after CAP treatment of up to 180 seconds. These results might indicate that severe apoptosis is not an early feature of CAP treatment in glioma cells. This observation is in line with several reports about the treatment of glioma cells, as it seems to be difficult to induce profound apoptosis in these cells rather than senescence [37],[38],[39]. We therefore studied the cell cycle distribution after CAP treatment. The observed robust cell cycle arrest in G2/M-phase (fig. 4) revealed to be the major effect of CAP treatment in glioma cells and was found in all treated cell lines. Noteworthy, also cells with unfavorable MGMT status responded to CAP treatment with cell cycle arrest. We further investigated for the effects of CAP on glioma clonogenicity. CAP treatment resulted in a significantly reduced clonogenicity in all treated cell lines, culminating in a complete loss of clonogenicity in LN18 cells with unfavorable MGMT status after 120 seconds of treatment (fig. 5). LN229 cells with favorable MGMT status demonstrated reduced clonogenicity after treatment with 50 µM TMZ, which was achieved to the same value by CAP treatment of 120 seconds. TMZ treatment alone of LN18 cells reduced the clonogenicity to a minor content compared to CAP treatment, whereas CAP treatment of 120 seconds was able to completely suppress clonogenicity. Our results on clonogenicity further confirm the decisive anti-cancer effect of CAP on glioma cells *in vitro*.

To our knowledge, this is the first report to show synergistic effects of the combined treatment with CAP and the chemotherapeutic TMZ on tumor cell growth and cell cycle distribution. Treated cells showed a significant reduction of the cell viability when treated for 30 seconds with CAP in combination with administration of TMZ (50 µM, 100 µM or 200 µM) in comparison to cells that were only treated with TMZ (fig. 6). For all tested cell lines, combined treatment was significantly effective in reducing cell growth. Concomitant treatment with 30 seconds of CAP and TMZ was more effective than exclusive treatment with TMZ or CAP. The significant dosage was 60 seconds of CAP in combination with each TMZ concentration in the LN229 cells, while 30 seconds combined with each TMZ concentration was sufficient for significantly reducing cell growth in LN18 and U87MG cells. Notably, combined treatment with 30 seconds plus 100 µM of TMZ and combination of 30 seconds of CAP plus 50 µM of TMZ, respectively, revealed stronger inhibition compared to higher dosage of TMZ (200 µM and 100 µM, respectively) in the LN18 and U87MG cell lines. Thus, concomitant therapy with CAP and TMZ might significantly increase the effectiveness of TMZ both in cells with favorable and unfavorable MGMT status. Furthermore, cell cycle arrest after CAP treatment was found to be prominent in glioma cells that are unsusceptible towards treatment with TMZ for up to 200 µM *in vitro* (fig. 7 A). A remarkable induction of a cell cycle arrest in the G2/M-phase in the TMZ resistant cell line LN18 after CAP treatment of 60 seconds and longer was noticed. A similar arrest in this cell line was observed only after treatment with concentrations of TMZ about 500 µM, while in contrast the clinically relevant concentrations of TMZ in the cerebrospinal fluid of the patient range between 3 µM and 50 µM [37],[40],[41]. A combined treatment of 60 seconds of CAP and 50 µM TMZ led to a cell cycle arrest, the combination of 60 seconds of CAP with 100 µM or 200 µM achieved an even more distinct arrest (fig. 7 B).

The mechanism behind the effect of CAP on tumor cells remains unknown. The production of ROS and the modification of cell membranes are supposed to mediate CAP effects on cells. Nevertheless, our results suggest CAP as a promising tool for the treatment of glioma cells *in vitro*, as the exclusive treatment as well as the combined treatment with TMZ revealed a cytostatic effect on the treated cell lines. So far, there is no suggestion that CAP directly affects the MGMT pathway. Therefore, CAP effectiveness seems not necessarily to be limited to cells with unfavorable MGMT status. It is noteworthy that CAP treatment was effective in tumor cells showing resistance to alkylating agents. Patients suffering from GBM with unfavorable MGMT status demonstrate only minor effects when treated with TMZ. However, no alternative treatment exists for this particular subgroup of patients until today. These patients as well as patients that acquire glioma cell resistance to alkylating chemotherapy during treatment might benefit from the application of concomitant treatment with CAP. To date, no resistance towards CAP treatment has been reported, adverting CAP as a promising tool in cancer therapy.

Translation of *in vitro* results into clinical application is demanding and therefore further investigations of CAP effects on tumorigenic and non-tumorigenic brain cells as well as *in vivo* studies in eligible animal models need to be carried out. Other studies concerning CAP application in GBM reported an induction of cell cycle arrest and DNA damage followed by a subsequent induction of apoptosis in U87MG glioblastoma cells treated with microsecond pulsed plasma. In a subcutaneous xenograft animal model a stabilization of tumor volume was achieved by treatment with plasma [28]. Further development of custom-designed CAP sources for application of CAP during surgery, including a new developed endoscopic device [27], will open the way for medical applications. Certainly, our results on CAP for combined treatment of malignant gliomas, especially in tumors that are resistant to alkylating agents, are hopeful future prospects.

## Supporting Information

Figure S1 **Detection of cytotoxicity induced by CAP treatment.** LN18 glioma cells were CAP treated and 2 h, 24 h and 48 h later the release of LDH was measured using the Roche Cytotoxicity Kit. Treatment with 1% Triton x-100 served as the positive control.
(JPG)

Figure S2 **Detection of DNA fragmentation after CAP treatment in LN229 glioma cells.** LN229 glioma cells were CAP treated without medium for indicated times and after 1 h and 24 h, respectively, the Comet assay was performed. The cell viability was observed simultaneously by tryphan blue staining.
(JPG)

Figure S3 **Cell cycle analysis of LN18 cells after CAP treatment.** Flow cytometry was performed 24 h, 48 h and 72 h after CAP treatment for the indicated times.
(JPG)

Figure S4 **Cell cycle analysis of LN229 cells after CAP treatment.** Glioma cells were CAP treated and cell cycle analysis was performed 24 h, 48 h and 72 h afterwards.
(JPG)

File S1 **Material and methods.**
(DOCX)
