# Supplementary figures and images for: Restoration of Sensitivity in Chemo — Resistant Glioma Cells by Cold Atmospheric Plasma
Source: PLoS One. 2013 May 21;8(5):e64498. doi: 10.1371/journal.pone.0064498 (PMC3660344; doi:10.1371/journal.pone.0064498)

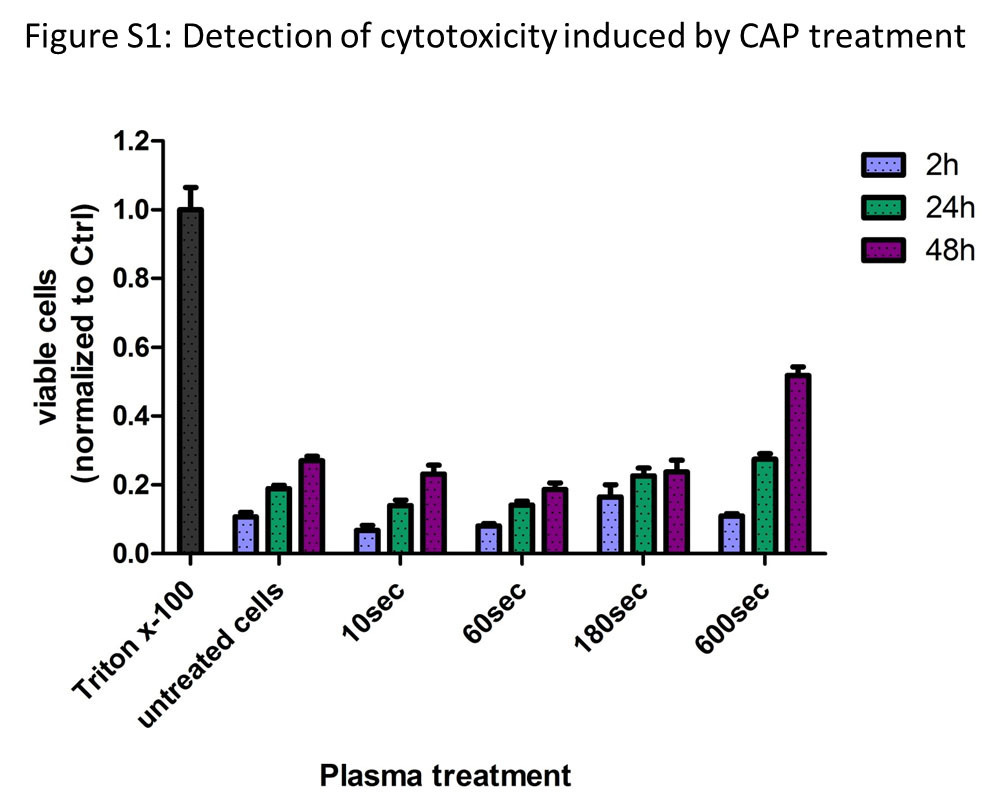

Supplement: Figure S1 — Detection of cytotoxicity induced by CAP treatment. LN18 glioma cells were CAP treated and 2 h, 24 h and 48 h later the release of LDH was measured using the Roche Cytotoxicity Kit. Treatment with 1% Triton x-100 served as the positive control. (JPG) [file pone.0064498.s001.jpg]

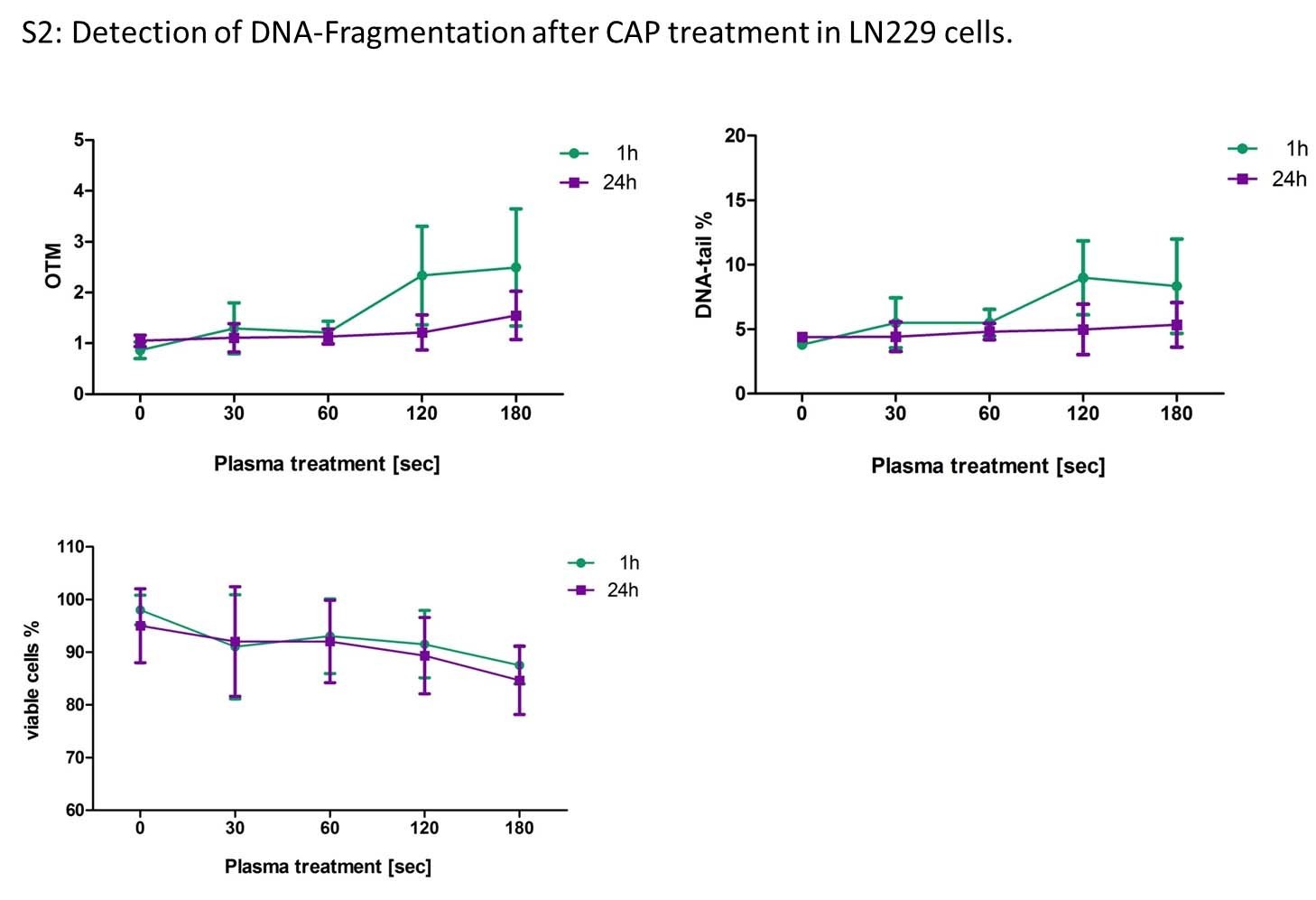

Supplement: Figure S2 — Detection of DNA fragmentation after CAP treatment in LN229 glioma cells. LN229 glioma cells were CAP treated without medium for indicated times and after 1 h and 24 h, respectively, the Comet assay was performed. The cell viability was observed simultaneously by tryphan blue staining. (JPG) [file pone.0064498.s002.jpg]

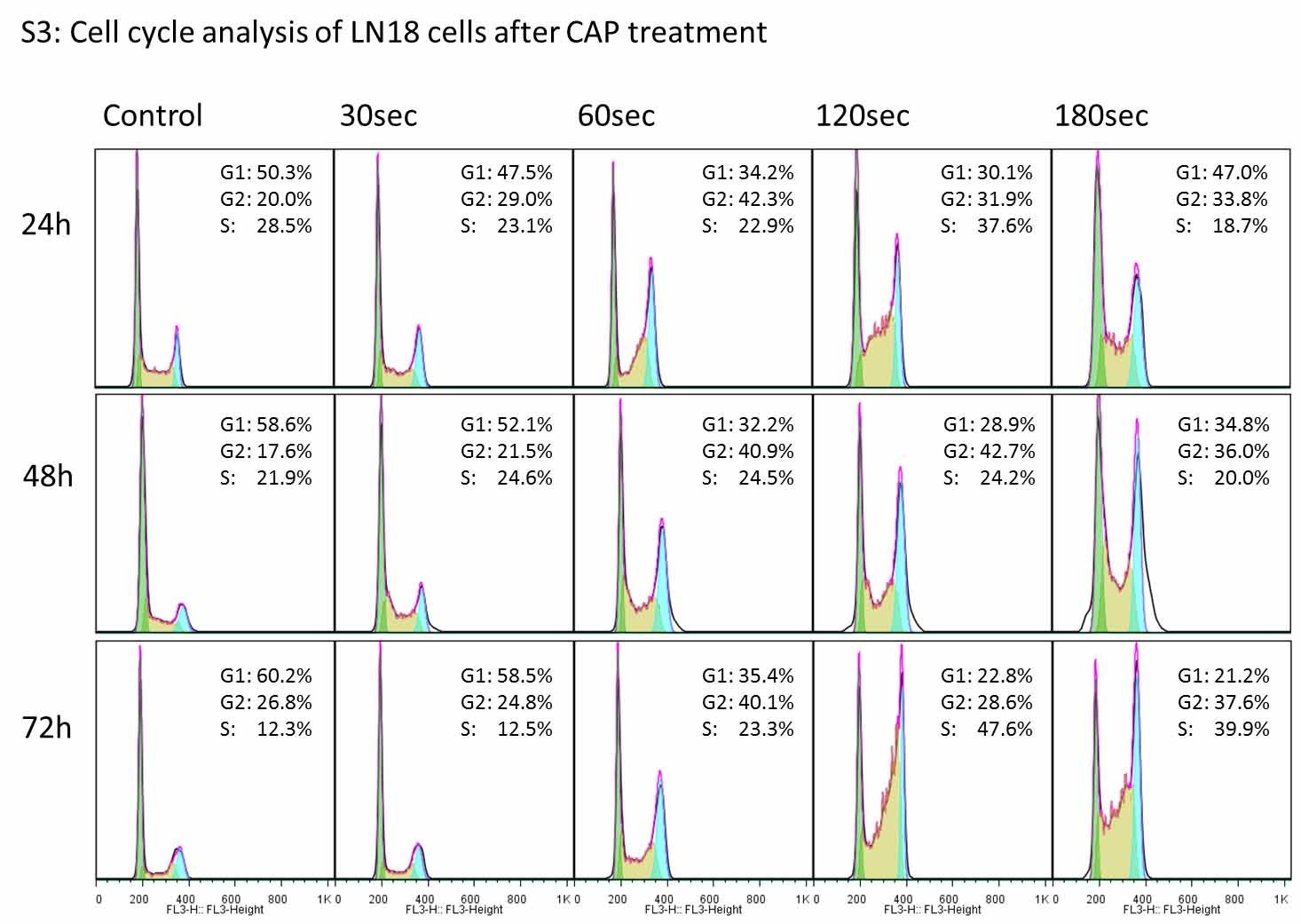

Supplement: Figure S3 — Cell cycle analysis of LN18 cells after CAP treatment. Flow cytometry was performed 24 h, 48 h and 72 h after CAP treatment for the indicated times. (JPG) [file pone.0064498.s003.jpg]

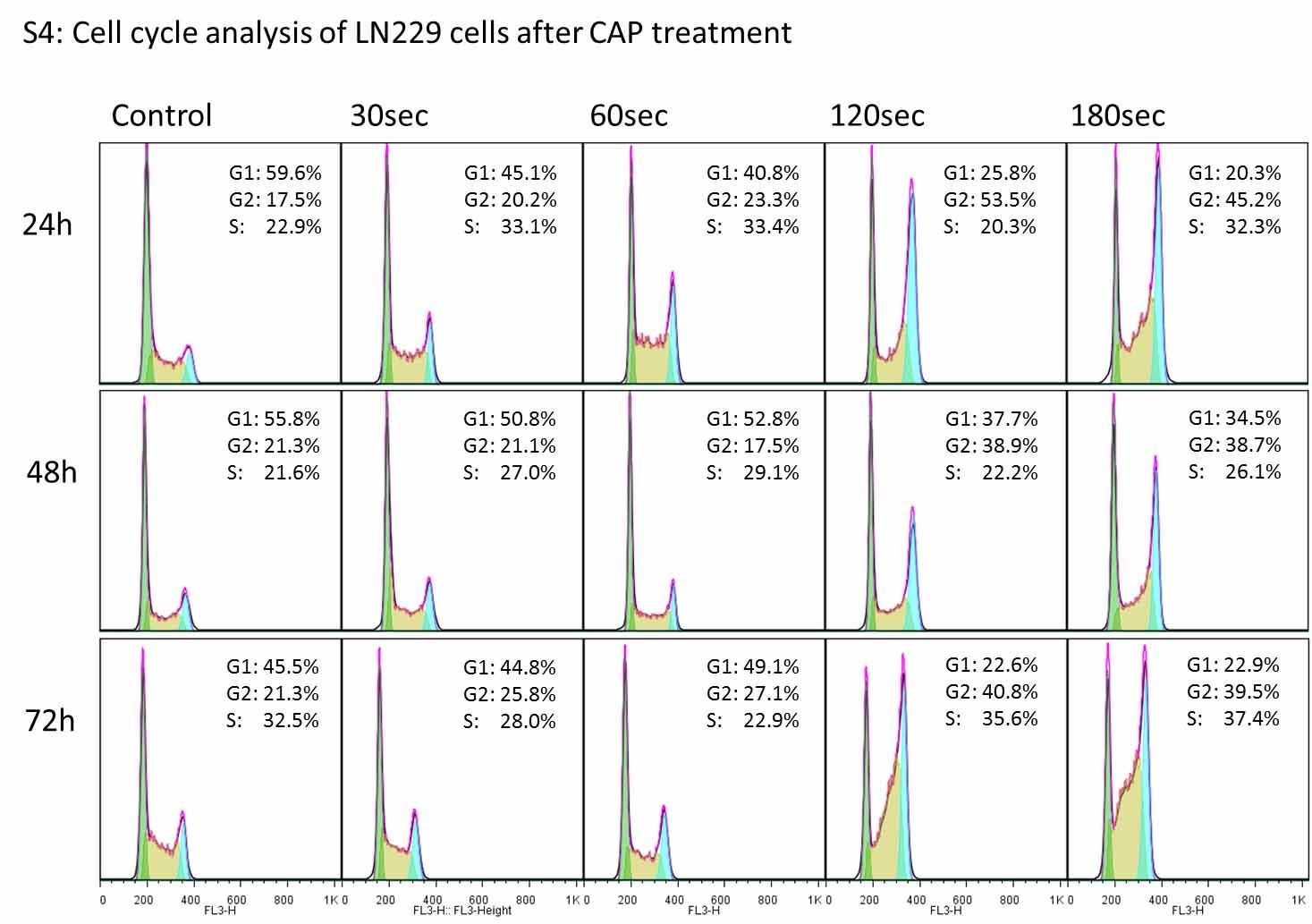

Supplement: Figure S4 — Cell cycle analysis of LN229 cells after CAP treatment. Glioma cells were CAP treated and cell cycle analysis was performed 24 h, 48 h and 72 h afterwards. (JPG) [file pone.0064498.s004.jpg]
