# Supplementary material for: Restoration of Sensitivity in Chemo — Resistant Glioma Cells by Cold Atmospheric Plasma
Source: PLoS One. 2013 May 21;8(5):e64498. doi: 10.1371/journal.pone.0064498 (PMC3660344; doi:10.1371/journal.pone.0064498)
Supplement: File S1 — Material and methods. (DOCX) [file pone.0064498.s005.docx]

**Material and methods**

Cytotoxicity

Cells were CAP treated without medium for the indicated times and fresh medium was added immediately after the treatment. LDH assay kit (Roche, Grenzach-Wyhlen, Germany) was performed according to the manufactures guidelines 2 h, 24 h and 48 h after CAP treatment. Treatment with 1% of Triton x-100 served as a positive control.

Comet Assay

Cell pellets were resuspended in 75 µl of 0.7 % low-melting agarose (Biozym, Hameln, Germany) and applied to slides frosted at the edges and covered with 0.5 % normal melting agarose (Biozym, Hameln, Germany). For cell lysis the slides were placed in alkali solution for 1 h (10 % DMSO, 1 % Triton-X, 2.5 M NaCl, 10 mM Trizma-Base, 100 mM Na2EDTA, and 1 % N-lauroylsarcosine sodium salt). Prior to electrophoresis, the slides were placed in a horizontal gel electrophoresis chamber (Renner, Dannstadt, Germany) and incubated with alkaline buffer solution containing 300 mM NaOH and 1 mM Na2EDTA at pH 13.2. After 20 min of DNA unwinding, electrophoresis was started at 0.8 V/cm and 300 mA and run for 20 min. Following neutralisation (Trisma base, 400mM, pH 7.5;Merck Germany) fluorescent DNA staining was performed with 75 µl ethidium bromide (Sigma; [51 µl]). Slides were analysed with a DMLB microscope (Leica, Bensheim, Germany). 80 cell nuclei per slide (2 slides per each CAP treatment) were selected with random pattern and digitized with the monochrome CCD camera (Cohu Inc., San Diego, CA, USA). Migration was measured by the image analysis software Komet++ (Kinetic Imaging, Liverpool, UK) using the % of DNA in tail and the Olive Tail Moments (OTM). The OTM represents the multiplication of the relative amount of DNA in the tail with the median migration distance. The % tail DNA is a measure of the relative fluorescent intensity in the head and tail. An OTM above 2 is widely accepted as DNA damage.
